# Trends and Characteristics in Marijuana Use Among Public School Students — King County, Washington, 2004–2016

**DOI:** 10.15585/mmwr.mm6839a3

**Published:** 2019-10-04

**Authors:** Myduc Ta, Lindsey Greto, Kaylin Bolt

**Affiliations:** ^1^Assessment, Policy Development & Evaluation Unit, Public Health—Seattle & King County, Washington; ^2^Youth Marijuana Prevention and Education Program, Chronic Disease & Injury Prevention Division, Public Health—Seattle & King County, Washington.

Use of marijuana at an early age can affect memory, school performance, attention, and learning; conclusions have been mixed regarding its impact on mental health conditions, including psychosis, depression, and anxiety (*1*–*3*). Medical marijuana has been legal in Washington since 1998, and in 2012, voters approved the retail sale of marijuana for recreational use to persons aged ≥21 years. The first retail stores opened for business in July 2014. As more states legalize marijuana use by adults aged ≥21 years, the effect of legalization on use by youths will be important to monitor. To guide planning of activities aimed at reducing marijuana use by youths and to inform ongoing policy development, Public Health—Seattle & King County assessed trends and characteristics of past 30–day marijuana use among King County, Washington, public school students in grades 6, 8, 10, and 12. This report used biennial data for 2004–2016 from the Washington State Healthy Youth Survey. Among grade 6 students there was a decreasing trend in self-reported past 30–day marijuana use from 2004 to 2016, while the percentage of grade 8 students who had used marijuana during the past 30 days did not change during that period. Among students in grades 10 and 12, self-reported past 30–day use of marijuana increased from 2004 to 2012, then declined from 2012 to 2016. In 2016, the percentage of students with past 30–day marijuana use in King County was 0.6% among grade 6, 4.1% among grade 8, 13.9% among grade 10, and 25.5% among grade 12 students. Among grade 10 students, 24.0% of past 30–day marijuana users also smoked cigarettes, compared with 1.3% of nonusers. From 2004 to 2016 the prevalence of perception of great risk of harm from regular marijuana use decreased across all grades. Continued surveillance using consistent measures is needed to monitor the impact of marijuana legalization and emerging public health issues, given variable legislation approaches among jurisdictions.

The Healthy Youth Survey is a school-based, anonymous, self-administered, cross-sectional survey conducted in the fall of even-numbered years in Washington public schools.* Schools with grades 6, 8, 10, and 12 are randomly selected using a clustered sampling design. Schools not selected for the state sample also can choose to participate in the survey. The survey measures risk behaviors, attitudes, and factors that contribute to youth health and safety, including alcohol, marijuana, tobacco, and other drug use; behaviors that result in unintentional and intentional injuries (e.g., violence); dietary behaviors, and physical activity.

This analysis used data from all participating schools, both sampled and nonsampled, representing all 19 King County school districts for biennial survey years 2004 through 2016 (the most currently available year of data at the time of analysis). King County is the largest metropolitan county in the state. Local jurisdictions have authority to regulate land uses and can impose additional time, place, and manner-of-use restrictions on state licensed businesses; thus, considerable variation in the availability of and restrictions on retail marijuana exists across the 39 cities in King County, including Seattle.

Survey response rates varied by grade and survey year, with higher rates in more recent surveys.^†^ During 2004–2016, King County response rates ranged from 60%–80% for grades 6 and 8; 50%–70% for grade 10; and 40%–50% for grade 12. For the 2016 survey, response rates for King County were 80% for grades 6 and 8, 70% for grade 10, 40% for grade 12, and 67% for all grades combined.

Data representing substance use, perception of great risk of harm, risky behaviors, and factors associated with marijuana use were categorized dichotomously. Past 30–day marijuana use was considered use on 1 or more days during the past 30 days. Perceived great risk of harm associated with regular marijuana use (more than one or two times per week) was categorized dichotomously as great risk versus all other options combined (moderate, slight, and no perceived risk). Past 30–day use of alcohol, cigarettes, and electronic cigarettes/vape pens was considered use on 1 or more days in the past 30 days, past 30–day risky driving and riding behaviors,^§^ were considered one or more occurrences during the past 30 days and past binge drinking^¶^ was over a 2-week period. 

Dichotomous factors generally reported to be associated with other substance use (*4*) were examined for marijuana use; these factors included whether students’ parents had talked about not using marijuana, use by one or more best friends or by a member in the youth’s household, and having been bullied one or more times in the past month.** Stata survey software (version 13; StataCorp) was used to generate percentage estimates and corresponding 95% confidence intervals (CIs). To account for differential participation among school districts across survey years, percentage estimates were weighted to the school district total enrollment by grade and sex, with the final weights adjusted to sum to the county total public school enrollment by grade and sex. Joinpoint trend analysis software (https://surveillance.cancer.gov/joinpoint/) was used to evaluate statistical significance of trends in survey-weighted percentage estimates by grade and sex. Analyses of trends by sex and examination of factors associated with past 30–day use were restricted to grade 10 students as a result of grade-specific sampling and the need for adequate response rates to accommodate a robust analysis.

During 2004–2016, the prevalence of reported past 30–day marijuana use was lowest among students in grade 6 and increased with school grade level (Figure 1). In 2016, past 30–day marijuana use was reported by 0.6% (CI = 0.4–0.7) of grade 6 students, 4.1% (CI = 3.5–4.8) of grade 8 students, 13.9% (CI = 12.6–15.3) of grade 10 students, and 25.5% (CI = 23.7–27.4) of grade 12 students in King County. Among students in grade 6, past 30–day marijuana use declined significantly, from 1.3% in 2004 to 0.6% in 2016. There was no statistically significant trend among students in grade 8; however, among students in grades 10 and 12, past 30–day use increased from 2004 to 2012, and then declined. Across all grades, the percentage of students reporting great risk of harm from regular marijuana use declined over the survey period, with the lowest perceived great risk of harm reported among older students in all years. In 2016, 26.7% (CI = 25.0–28.5) of students in grade 12 perceived great risk of harm from regular marijuana use, whereas 53.3% (CI = 50.5–56.1) reported this perception in 2004.

**FIGURE 1:**
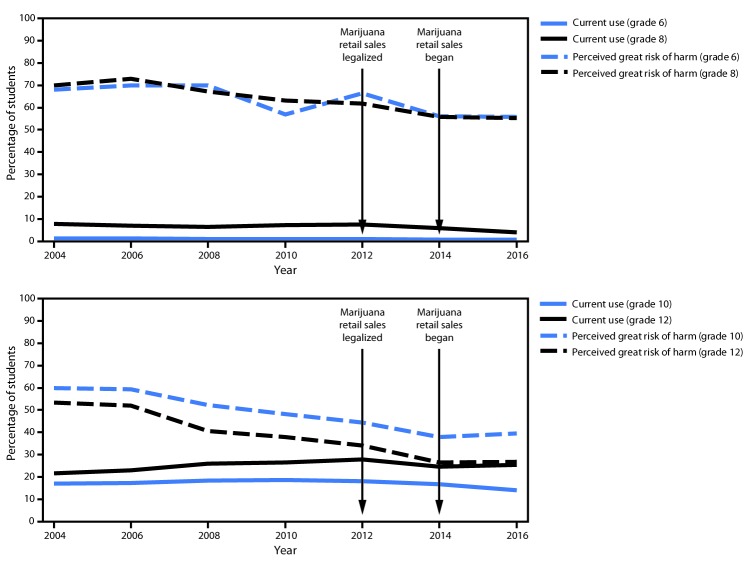
Percentage of students with past 30–day (current) marijuana use* and their perception of great risk of harm^†^ associated with marijuana use, by school grade — Healthy Youth Survey, King County, Washington, 2004–2016 * Significant decreasing trend (p<0.05) in past 30-day marijuana use for grade 6. Change in trend starting in 2012 for grades 10 and 12. ^†^ Significant decreasing trend (p<0.05) in perception of great risk of harm from marijuana use for all grades.

Among male students in grade 10, past 30–day marijuana use increased from 17.6% in 2004 to 21.4% in 2010 and subsequently declined to 13.5% in 2016 (Figure 2). Among female students in grade 10, there was no change in the prevalence of past 30–day use, which remained approximately 16% during this period. In 2016, there was no significant difference in past 30–day marijuana use between male and female students in grade 10.

**FIGURE 2:**
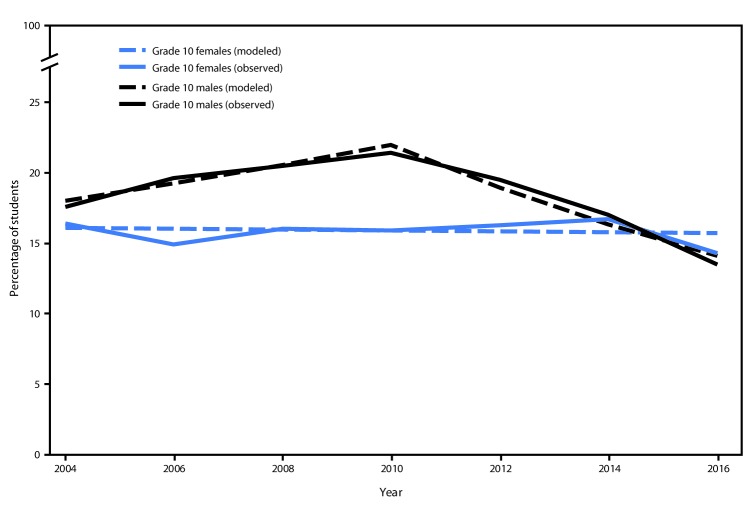
Percentage of students who were past 30–day (current) marijuana users among grade 10 students, by sex — Healthy Youth Survey, King County, Washington, 2004–2016* * Significant (p<0.05) change in trend among male grade 10 students starting in 2010.

Among past 30–day marijuana users in grade 10, 42.8% reported living with someone who uses marijuana, 88.5% reported having at least one best friend who used marijuana, and 26.3% reported having been bullied at least once in the past 30 days; these prevalences were higher than those among grade 10 nonusers (12.8%, 28.3%, and 16.5%, respectively) (Table). Among grade 10 marijuana users, 92.5% reported that it was not very hard to obtain marijuana, compared with 56.7% of nonusers. No parental discussion about marijuana during the past year was reported by similar percentages of past 30–day marijuana users (39.2%) and nonusers (39.8%).

**TABLE:** Prevalence of marijuana use among 10th grade public school students in the past 30 days and prevalence ratios between marijuana users and nonusers, by selected characteristics (N = 14,055) — Healthy Youth Survey, King County, Washington, 2016

Characteristic	Marijuana use in past 30 days*		
	Yes (n = 1,949), % (95% CI)	No (n = 12,106), % (95% CI)	Prevalence ratio marijuana users/ nonusers (95% CI)
**Overall**	**13.9 (12.6–15.3)**	**86.1 (84.7–87.4)**	**N/A**
**Individual/Family factors^†^**			
Household marijuana use^§^	42.8 (39.2–46.5)	12.8 (11.4–14.4)	3.3 (2.9–3.9)
Parents have not talked about not using marijuana	39.2 (36.1–42.3)	39.8 (38.1–41.4)	1.0 (0.9–1.1)
≥1 best friend who used marijuana	88.5 (85.7–90.8)	28.3 (26.1–30.6)	3.1 (2.9–3.4)
Perceived great risk of harm from regular marijuana use	8.1 (6.6–9.8)	45.0 (42.5–47.5)	0.18 (0.15–0.22)
Not very hard to get marijuana	92.5 (90.8–93.9)	56.7 (54.8–58.6)	1.6 (1.6–1.7)
At academic risk^¶^	35.5 (31.4–39.7)	15.1 (13.2–17.2)	2.4 (2.1–2.7)
Bullied ≥1 time in past 30 days	26.3 (23.8–28.9)	16.5 (15.5–17.6)	1.6 (1.4–1.8)
Driving within 3 hours of using marijuana at least once in the past month	36.0 (31.7–40.6)	N/A	N/A
Rode in car at least once in the past month with driver who has used marijuana	60.8 (56.7–64.7)	6.8 (5.8–7.9)	9.0 (7.8–10.3)
Additional substance use**			
Alcohol	67.0 (63.9–70.0)	10.3 (9.5–11.2)	6.5 (6.0–7.0)
Cigarette smoking	24.0 (21.4–26.7)	1.3 (1.1–1.5)	18.9 (16.1–22.0)
Electronic cigarettes/Vape pens	43.0 (37.4–48.8)	4.0 (3.3–4.7)	10.9 (9.3–12.7)
Binge drinking^††^	43.5 (40.4–46.7)	3.7 (3.3–4.1)	11.9 (10.5–13.5)
Any substance use^§§^	88.6 (86.6–90.3)	22.1 (20.6–23.7)	4.0 (3.7–4.3)

**Abbreviations:** CI = confidence interval; N/A = not applicable.

* All estimates are survey weighted to reflect total county public school enrollment by grade and sex; prevalence ratios are unadjusted comparing marijuana users and nonusers for individual or family factors and additional substance use.

^†^ Marijuana users and nonusers who reported a given individual or family factor. Denominators for categories listed might be less than total marijuana users and nonusers because some respondents only responded to the marijuana use question.

^§^ Lives with someone who uses marijuana.

^¶^ Grades of “C” or lower.

** Marijuana users and nonusers who reported using other substances on 1 or more days in the past 30 days. Denominators for categories listed might be less than total marijuana users and nonusers because some respondents only responded to the marijuana use question.

^††^ Consumed five or more alcoholic drinks in a row during the preceding 2 weeks.

^§§^ Reported use of alcohol, cigarette smoking, or e-cigarette/vape pen on one or more days in the past 30 days, or reported binge drinking.

Among grade 10 students, prevalence of past 30–day use of other substances was four times higher among those who had used marijuana in the past 30 days than among those who had not. Among marijuana users, the prevalences of past 30–day use of other substances were as follows; alcohol (67.0%), cigarettes (24.0%), e-cigarettes or vape pens (43.0%), and of binge drinking (43.5%), compared with 10.3%, 1.3%, 4.0%, and 3.7% among nonusers, respectively. Among grade 10 marijuana users, 36% reported driving within 3 hours of using marijuana at least once in the past month.

## Discussion

Despite legalization of the retail sale of marijuana to adults in Washington in 2012, evidence from the biennial Washington State Healthy Youth Survey indicates that the prevalence of past 30–day marijuana use among students in grades 10 and 12 began to decline that year. The decline continued in 2016 among grade 10 students and did not change significantly among grade 12 students. This decline or absence of change in youth marijuana use after legalization of retail sales to adults is consistent with trends reported in Colorado and Oregon,^††^ states that legalized adult retail sales of marijuana in 2013 and 2014, respectively. However, causality of the observed decrease in youth use following retail sale legalization cannot be inferred, because effects might be delayed and this report does not include data from the timeframe that would capture the more recent surge in e-cigarette use by youth and the use of tetrahydrocannabinol (THC) within electronic cigarette (e-cigarette) devices. Although the relationship between legal adult recreational use and youth use is not well understood, two possible reasons for the observed decline in youth use include reduction of illicit market supply through competition^§§^ and loss of novelty appeal among youths. Furthermore, it would be important to monitor the long-term role legalization might play to foster a permissive use environment given observed strong associations with use and individual and family factors that influence youth use.

Before initiation of retail marijuana sales in Washington in 2014, the statewide prevalence of use among grade 10 students had not changed significantly since 2002, although reported statewide use prevalence in 2016 was higher among students identifying as non-Hispanic American Indian/Alaska Native and Hispanic than among non-Hispanic white and non-Hispanic Asian students (*5*). Among grade 10 King County students, past 30–day marijuana use by male students has been decreasing since 2010, while the prevalence among female students has not changed. Continued monitoring is necessary to observe how local trends among males change over time. The narrowing of the sex difference gap reflects national trends (*6*) and suggests that female users might benefit from tailored prevention messages informed by an understanding of reasons for use.

Although overall youth rates of smoking and alcohol are declining nationally (*7*), the prevalence of any substance use, including alcohol, cigarettes, or vape pens, was four times higher among grade 10 past 30–day marijuana users than among nonusers. Statewide data from 2016 also show similar higher prevalence of household, peer and individual factors associated with youth substance use among grade 10 marijuana users than nonusers (https://www.askhys.net/library/2016/RecentMarijaunaUseGr10.pdf). Findings from a 2017 survey of Canadian residents aged 15–24 years found that marijuana users were significantly more likely to be past 30-day e-cigarette users, compared with nonusers (*8*). Polysubstance use and driving after using marijuana or riding in a car driven by someone who had used marijuana recently are public health issues that are important to monitor. Educational campaigns conveying health risk of marijuana use should also address impaired driving, in light of experimental data showing deteriorating control with increasing task complexity and increased risk for involvement in a motor vehicle crash (*9*).

The findings in this report are subject to at least six limitations. First, these data predate the recent reported increase in youth e-cigarette use and the use of THC in the newest generation of e-cigarette devices. The marijuana use question does not explicitly define use by method and estimates of youth marijuana use might be underestimated if respondents did not consider vaping or edible consumption of marijuana products when responding to the question. Second, data are from public school students only and might not be generalizable to all youths in this age group. Students who might be at higher risk might not be in school; it is estimated that 95.3% of King County residents aged 14–18 years are in school.^¶¶^ Third, survey participation is voluntary, and responses are based on self-report, which can be subject to recall or response bias. Fourth, these estimates might differ from other state or nationally representative youth health–surveillance systems, in part because of survey methods, age of participants, survey setting, and period during the year the survey was conducted. Fifth, local historical data for youth marijuana use before 2004 are not available, and the effects of medical marijuana legalization, which occurred in 1998, on use by youths is unknown. Finally, binge drinking is framed as five or more drinks in a row during the preceding 2 weeks for both males and females and would likely underestimate excessive alcohol consumption among females compared with using a sex-specific four-drink threshold (*10*).

The national goals for substance use set by Healthy People 2020*** include a target of 6% for youths aged 12–17 years with past 30–day marijuana use, and progress toward this target requires evidence-based interventions and policies for preventing and treating substance use and abuse among youths. Although some cross-cutting interventions addressing adolescent health are presented in the Community Preventive Task Force’s Community Guide,^†††^ there currently is no specific category for marijuana use, as there is for alcohol and tobacco. The National Registry of Evidence-based Programs and Practices,^§§§^ a project of the federal Substance Abuse and Mental Health Services Administration, might be a potential alternative source for strategies that reduce marijuana use and prevent associated harms, but these strategies might not be sufficient for states with newly legalized retail marketplaces. In light of the limited evidence base, there is a need to identify individual, relationship, community, and societal determinants of youth substance use that would allow development of broad-based risk-reduction strategies. Continued surveillance would benefit from having a set of standard measures across jurisdictions to monitor the health impacts of retail marijuana sale legalization among states.

**Summary**

**What is already known about this topic?**

Youth marijuana use can have adverse health outcomes. However, reports from Colorado, Oregon, and Washington indicate no statewide increase in youth marijuana use following retail legalization for adults.

**What is added by this report?**

Following 2012 legalization of retail marijuana sale to adults in Washington, past 30–day marijuana use decreased or remained stable through 2016 among King County students in grades 6, 8, 10, and 12. Among grade 10 students, the decline in use occurred among males while the rate among females remained steady. Use of alcohol or other substances was four times as frequent among marijuana users as among nonusers.

**What are the implications for public health practice?**

Understanding reasons for youth marijuana use, particularly among females, might help inform policy, strategies, and educational campaigns.

## Footnotes

*
https://www.doh.wa.gov/DataandStatisticalReports/DataSystems/HealthyYouthSurvey/TechnicalNotes .

† During the analysis period, two south King County school districts had inconsistent participation: one district did not participate in 2008 and 2010, and the other district did not participate in 2004. A third school district has not participated since 2004 because of small enrollment (<40 students districtwide).

§ Driving within 3 hours of using marijuana and risky riding defined as riding with a driver who had used marijuana.

¶ Consuming five or more drinks in a row in the past 2 weeks.

** https://www.drugabuse.gov/publications/preventing-drug-abuse-among-children-adolescents/chapter-1-risk-factors-protective-factors and https://iprc.iu.edu/spf/docs/Risk%20and%20Protective%20Factors%20Associated%20with%20Youth%20Marijuana.pdf.

†† https://www.colorado.gov/cdphe/marijuana-health-report and https://www.oregon.gov/oha/PH/PREVENTIONWELLNESS/MARIJUANA/Documents/HB3400-Legislative-Report-Youth-Prevention-2017.pdf.

§§ https://www.wsipp.wa.gov/ReportFile/1670/Wsipp_I-502-Evaluation-and-Benefit-Cost-Analysis-Second-Required-Report_Report.pdf.

¶¶ Based on analysis of 2012–2016 U.S. Census Bureau American Community Survey Public Use Microsample data, 95.3% (95% CI = 94.4%–96%) of the King County population aged 14–18 years are in school; 98% (95% CI = 97.3%–98.6%) of the King County population aged 15–16 years (grade 10) are in school.

*** https://www.healthypeople.gov/2020/topics-objectives/topic/substance-abuse.

††† https://www.thecommunityguide.org; https://www.healthypeople.gov/2020/tools-resources/Evidence-Based-Resources.

§§§
https://www.samhsa.gov/ebp-resource-center .
